# Supplementary material for: Enhanced fibrinolysis detection in a natural occurring canine model with intracavitary effusions: Comparison and degree of agreement between thromboelastometry and FDPs, D-dimer and fibrinogen concentrations
Source: PLoS One. 2019 Nov 14;14(11):e0225089. doi: 10.1371/journal.pone.0225089 (PMC6855488; doi:10.1371/journal.pone.0225089)
Supplement: Attachment — Submitted filename: Response to Reviewers.docx [file pone.0225089.s002.docx]

**RE: Manuscript PONE-D-19-15389**

Enhanced fibrinolysis detection in a natural occurring canine model with intracavitary effusions: comparison and degree of agreement between thromboelastometry and FDPs, D-dimer and fibrinogen concentrations

Dear Dr. Pablo Garcia de Frutos,

The authors would like to thank You and the Reviewers for the time spent and for their constructive comments in attempt to improve our manuscript.

A number of changes (listed below) have been made to the current manuscript to incorporate the suggestions you made and to address the comments of the Reviewers. We hope that these are satisfactory to allow for publication. Belows follow our reply to your 3 specific requests.

**1)** The authors should include healthy values for comparison obtained in the same conditions.

**Authors’ reply:** To give an idea for comparison to healthy values, reference intervals (obtained from healthy animals) for all the parameters studied were included (and still are) in the result section of our paper. On the other hand, if the Editor meant that we should include a healthy control population, as requested from Reviewer #2, we cannot adhere to this request because it would heavily damage the validity of our study (please see below).

Case–control is a type of epidemiological observational study where subjects are *observed* in order to determine both their exposure and outcome status. A common misconception among investigators is that case-control design is perceived as a comparison between a group of diseased individuals versus a group of “normal” (i.e., disease-free) individuals. This conceptualization of the case-control design is inconsistent with the actual goal of control group selection, which is to provide a representative sample of the risk factors distribution among the population at risk. Therefore, following the current epidemiology textbooks indications and international guidelines on observational studies, we have included as a control population a group of sick dogs as much as possible similar to the specific diseased group (cases), but without the specific outcome of interest that we wanted to study (i.e., the presence of intracavitary effusion). On the other hand, a control group of healthy animals would have weakened the study, because different for too many aspects from a diseased group and not even representative of the general population. Selection of healthy subjects has been heavily criticized in the modern literature and the so-called “healthy patient bias” has been coined (REFERENCES follow).

1) Bloom MS, Schisterman EF, Hediger ML. Selecting controls is not selecting “normals”: design and analysis issues for studying the etiology of polycystic ovary syndrome. Fertil Steril. 2006;86(1):2-12

2) Lewallen S, Courtright P. Epidemiology in practice: case-control studies. Comm Eye Health 1998;11:57-58

3) Wacholder JK, Silverman JS, McLaughlin JK, Mandel JS. Selection of controls in case-control studies. II. Types of Controls. Am J Epidemiol; 1992, 135:1029-1041

4) Shrank WH, Patrick AR, Brookhart A. Healthy user and related biases in observational studies of preventive interventions: a primer for physicians. J Gen Intern Med, 2011;26(5):546-550

**2)** Further, the presence of bleeding in both groups of studied animals makes difficult to interpret the results of the ROTEM analysis.

**Authors’ reply:** To address your concern additional statistical analysis excluding bleedings dogs from both groups were performed as requested. The additional analyses showed that bleeding is not the solely driving of our finding. In fact, excluding these dogs none of the results changed. Please see the 'Revised Manuscript with Track Changes’ at:

• Materials and methods lines: 214-220

• Results: lines 269-271; 279-283; 357-360; 364-367; 372-374; Table 1, 3, 4, and 5.

• Discussion: 427–430; 462–462; 484–485; 520–521.

**3)** The presentation of the results in the figures should be optimized. The graphs could be combined in one figure with different panels. Note that there is a typo in figure one ("intracavitay").
**Authors’ reply:** Following your instruction the 5 figures have been combined in one figure with 5 panels. The typo mistake has been corrected.

Kind regards,

Andrea Zoia, DVM, Cert SAM, Dip ECVIM-CA

(Corresponding Author)

PS: If you have any further questions or comments, please do not hesitate to contact me.

**Journal requirements:**

When submitting your revision, we need you to address these additional requirements.

**1.** Please ensure that your manuscript meets PLOS ONE's style requirements, including those for file naming. The PLOS ONE style templates can be found at

<http://www.journals.plos.org/plosone/s/file?id=wjVg/PLOSOne_formatting_sample_main_body.pdf> and <http://www.journals.plos.org/plosone/s/file?id=ba62/PLOSOne_formatting_sample_title_authors_affiliations.pdf>

**Authors’ reply:** following the above PDFs instructions, numerous changes have been made to the article’s format. If we still do not meet Plos One’s style requirements we would be grateful to receive more specific suggestions.

**2.** We noticed you have some minor occurrence of overlapping text with the following previous publications, which needs to be addressed:

-'Hemostatic findings of pleural fluid in dogs and the association between pleural effusions and primary hyperfibrino(geno)lysis: A cohort study of 99 dogs', <https://doi.org/10.1371/journal.pone.0192371>
-'Evaluation of rotation thrombelastography for the diagnosis of hyperfibrinolysis in trauma patients', <https://doi.org/10.1093/bja/aen083>

In your revision ensure you cite all your sources (including your own works), and quote or rephrase any duplicated text outside the methods section. Further consideration is dependent on these concerns being addressed.

**Authors’ reply:** Overlapping test in figure caption of this paper with our previous work “'Hemostatic findings of pleural fluid in dogs and the association between pleural effusions and primary hyperfibrino(geno)lysis: A cohort study of 99 dogs” was present and it has been drastically reduced/eliminated. There does remain some overlap between this article and the above article only for the author affiliation and for the references, which from our understanding should not constitute an issue.

Small overlapping test in the introduction of this paper with the 'Evaluation of rotation thrombelastography for the diagnosis of hyperfibrinolysis in trauma patients' had been rephrased as requested.

**3.** Thank you for stating the following in the Competing Interests/Financial Disclosure* (delete as necessary) section:

"The author(s) received no specific funding for this work"

We note that one or more of the authors are employed by a commercial company: San Marco Veterinary Clinic, Padua, Italy.

a) Please provide an amended Funding Statement declaring this commercial affiliation, as well as a statement regarding the Role of Funders in your study. If the funding organization did not play a role in the study design, data collection and analysis, decision to publish, or preparation of the manuscript and only provided financial support in the form of authors' salaries and/or research materials, please review your statements relating to the author contributions, and ensure you have specifically and accurately indicated the role(s) that these authors had in your study. You can update author roles in the Author Contributions section of the online submission form.

Please also include the following statement within your amended Funding Statement.
“The funder provided support in the form of salaries for authors [insert relevant initials], but did not have any additional role in the study design, data collection and analysis, decision to publish, or preparation of the manuscript. The specific roles of these authors are articulated in the ‘author contributions’ section.”

If your commercial affiliation did play a role in your study, please state and explain this role within your updated Funding Statement.

b)  Please also provide an updated Competing Interests Statement declaring this commercial affiliation along with any other relevant declarations relating to employment, consultancy, patents, products in development, or marketed products, etc.

Within your Competing Interests Statement, please confirm that this commercial affiliation does not alter your adherence to all PLOS ONE policies on sharing data and materials by including the following statement: "This does not alter our adherence to PLOS ONE policies on sharing data and materials.” (as detailed online in our guide for authors <http://journals.plos.org/plosone/s/competing-interests>) . If this adherence statement is not accurate and there are restrictions on sharing of data and/or materials, please state these. Please note that we cannot proceed with consideration of your article until this information has been declared.

Please include both an updated Funding Statement and Competing Interests Statement in your cover letter. We will change the online submission form on your behalf.

Please know it is PLOS ONE policy for corresponding authors to declare, on behalf of all authors, all potential competing interests for the purposes of transparency. PLOS defines a competing interest as anything that interferes with, or could reasonably be perceived as interfering with, the full and objective presentation, peer review, editorial decision-making, or publication of research or non-research articles submitted to one of the journals. Competing interests can be financial or non-financial, professional, or personal. Competing interests can arise in relationship to an organization or another person. Please follow this link to our website for more details on competing interests: <http://journals.plos.org/plosone/s/competing-interests>

**Authors’ reply:** The San Marco Veterinary Clinic (Padua, Italy) did not play any role in the study nor altered our adherence to all PLOS ONE policies on sharing data and materials. An updated Funding Statement and a Competing Interests Statement has been included in the cover letter, as requested.

[Note: HTML markup is below. Please do not edit.]

Reviewers' comments:

Reviewer's Responses to Questions

**Comments to the Author**

1. Is the manuscript technically sound, and do the data support the conclusions?

The manuscript must describe a technically sound piece of scientific research with data that supports the conclusions. Experiments must have been conducted rigorously, with appropriate controls, replication, and sample sizes. The conclusions must be drawn appropriately based on the data presented.

Reviewer #1: Yes

**Authors’ reply:** none requested

Reviewer #2: Partly

**Authors’ reply:** please see our reply to your comments below.

2. Has the statistical analysis been performed appropriately and rigorously?

Reviewer #1: No

**Authors’ reply:** additional statistical analysis excluding dogs with bleeding has been added as requested (see our reply to your comments below).

Reviewer #2: N/A

**Authors’ reply:** none requested

**3.** Have the authors made all data underlying the findings in their manuscript fully available?

The [PLOS Data policy](http://www.plosone.org/static/policies.action#sharing) requires authors to make all data underlying the findings described in their manuscript fully available without restriction, with rare exception (please refer to the Data Availability Statement in the manuscript PDF file). The data should be provided as part of the manuscript or its supporting information, or deposited to a public repository. For example, in addition to summary statistics, the data points behind means, medians and variance measures should be available. If there are restrictions on publicly sharing data—e.g. participant privacy or use of data from a third party—those must be specified.

Reviewer #1: Yes

**Authors’ reply:** none requested

Reviewer #2: Yes

**Authors’ reply:** none requested

**4.** Is the manuscript presented in an intelligible fashion and written in standard English?

PLOS ONE does not copyedit accepted manuscripts, so the language in submitted articles must be clear, correct, and unambiguous. Any typographical or grammatical errors should be corrected at revision, so please note any specific errors here.

Reviewer #1: Yes

**Authors’ reply:** none requested

Reviewer #2: Yes

**Authors’ reply:** none requested

**5. Review Comments to the Author**

**Reviewer #1:**

**1)** This is a well written manuscript with well described methods and statistics. My major concern about this study is the heterogenous patient population, which I am concerned limits the value of the data analysis.

**Authors’ reply:** We thank the Reviewer to find our manuscript well written and with appropriate description on methods and statistics. To address your concern on the heterogenous patient population further statistical analysis excluding bleedings dogs from both groups were performed. For details see our reply to your point number 3.

**2)** The basic premise of the study is that patients with peritoneal and pleural effusions are more likely to have increased fibrinolysis than patients without effusions, but the authors present no rationale for this hypothesis. While they cite their previous works documenting traditional coagulation and fibrinolysis assays that support the concept, I am perplexed by the mechanisms that would underlay this finding, and the authors do not attempt to explain why a dog with right sided heart failure would develop hyperfibrinolysis.

**Authors’ reply:** We thank the Reviewer for this comment which allowed us to improve our article. In our previous works cited in the current article there is well explained mechanism that would justify why dogs with peritoneal and pleural effusions are more likely to have increased fibrinolysis than patients without effusions, including dogs with right sided heart failure. It is true that this information is not present in the current article. Therefore, we briefly summarized rationale for this hypothesis also in this article following your comment, leaving the references to acquire more in-depth details for the readers more interested in such pathophysiological information. Moreover further 12 references (references 35 to 46) have been added to support this hypothesis. Please see 'Revised Manuscript with Track Changes’ at lines 465-468.

**3)** Given that 10/23 dogs with effusions had hemoperitoneum, I am concerned that the findings in this study are driven largely by the dogs that bled. A statistical analysis (or at least descriptive statistics) showing how many of the dogs that demonstrated hyperfibrinolysis were hemoperitoneum cases and how many were not bleeding should be done to determine if all of these findings are driven by the bleeding dogs. I would argue that combining all of these types of disease processes into a single group makes these findings very difficult to interpret. Also having no data on the severity of the clinical signs and degree of shock in the bleeding patients makes it difficult to decide how to use the results of this study since pervious work has shown that the severity of shock is closely associated with viscoelastic measures of hyperfibrinolysis in dogs (Fletcher et al., JVECC, 2016). I worry that the small number of cases in this study and the heterogeneity of the group make the findings difficult to interpret and impossible to generalize. Without some sort of statistical analysis demonstrating that the hemoperitoneum cases are not solely driving these findings, I cannot endorse the conclusions of the authors.

**Authors’ reply:** We thank the Reviewer for this comment that allow us to improve our article. To address your concern additional statistical analysis excluding bleedings dogs from both groups were performed as requested. The additional analyses showed that bleeding is not the solely driving of our finding. In fact, excluding these dogs none of the results changed. Please see 'Revised Manuscript with Track Changes’ at:

• Materials and methods lines: 214-220

• Results: lines 269-271; 279-283; 357-360; 364-367; 372-374; Table 1, 3, 4, and 5.

• Discussion: 427–430; 462–462; 484–485; 520–521.

**4)** The classification scheme proposed to categorize fibrinolysis in these patients is interesting and potentially useful, but I have some concerns that with the very small number of dogs in this study, these numerous classifications are confusing and of questionable benefit in the study. The arbitrary cutoffs for the ROTEM and traditional parameters evaluated would need to be validated in a larger study, and I worry that this complex scheme detracts from the findings of the paper. I would suggest that using a more simplified scheme denoting patients as hyperfibrinolytic, with normal systemic fibrinolysis, and hypofibrinolytic would improve the clarity of this paper.

**Authors’ reply:** We thank the Reviewer to find the classification scheme proposed to categorize fibrinolysis in our patients interesting and potentially useful. Nevertheless, we realize that you have some concern with the very small number of dogs in this study being analysed in multiple subgroups. However, while in the descriptive statistics we have several groups (hypofibrinolysis, basal fibrinolysis, increased fibrinolysis, and hyerfibrinolys, with the latter two groups sometimes dived in primary and secondary) in our inferential statistics we used, to simplify the classification system, only 3 groups of fibrinolysis. In fact, hypo and basal fibrinolysis are always grouped together and for the same reasons we also kept together dogs with primary and secondary increased fibrinolysis and primary and secondary hyperfibrinolysis. These information are now better spelled/explained in the captions of tables 3, 4, 5, and 6.

Our cut-offs for the ROTEM and traditional parameters are not as “arbitrary” as you state. In fact, in part they are justify throughout the paper with biological explanations (i.e., fibrinogen) and in part they are in line with human and veterinary studies where fibrinolysis cut-offs are based on reference intervals. Nevertheless, we do agree with you that, as any new classification system, replication studies need to be performed to validate our proposed classification system.

Finally, we cannot adhere to your suggestion of using a more simplified scheme including only the hyperfibrinolytic, the normal fibrinolytic and the hypofibrinolytic state for two reasons:

a) It would change partially the target of our study. In fact, we also aim to try to differentiate patient with increased fibrinolysis that are at risk of bleeding (hyperfibrinolysis) from patient with increased fibrinolysis that are probably not at risk of bleeding (increased fibrinolysis). In addition, we also try to differentiate primary from secondary fibrinolysis. Unfortunately, we would not be able to do these types of differentiations with the scheme from you proposed.
b) As a LI60 of 100% essentially encompasses the normal range, some authors consider the ROTEM in essence unsuitable to define hypofibrinolysis (although this is done extensively in human’s trauma research). For this reason, we decided in our inferential statistics to combine the hypo and the basal fibrinolysis in a single group, leaving this distinction only for the descriptive statistics.

REFERENCE: Lisman T. Decreased Fibrinolytic Capacity in Cirrhosis and Liver Transplantation Outcomes. Liver Transpl. 2019 Mar;25(3):359-361.

**Reviewer #2:**

**1)** In this veterinary clinical study, the authors select two groups of animals from a large cohort considering the presence of cavitary effusion. Their aim is to study the fibrinolytic status of these animals in relation to the presence of pleural, peritoneal, or pericardial effusions by ROTEM technology. The study has several limitations that should be considered by the authors. They do not have a healthy control study group to compare their results on sick animals. The authors label their control group as "Sick dogs without intracavitary effusion". However, in both groups the animals are sick. It would be important to have an adequate healthy group for comparison.

**Authors’ reply:** To give an idea for comparison to a healthy population, reference intervals (obtained from healthy animals) for all the parameters studied were included (and still are) in the result section of our paper. On the other hand, a true healthy control group cannot be added, as requested from You, because it would heavily damage the validity of our study (please see below).

Case–control is a type of epidemiological observational study where subjects are *observed* in order to determine both their exposure and outcome status. A common misconception among investigators is that case-control design is perceived as a comparison between a group of diseased individuals versus a group of “normal” (i.e., disease-free) individuals. This conceptualization of the case-control design is inconsistent with the actual goal of control group selection, which is to provide a representative sample of the risk factors distribution among the population at risk. Therefore, following the current epidemiology textbooks indications and international guidelines on observational studies, we have included as a control population a group of sick dogs as much as possible similar to the specific diseased group (cases), but without the specific outcome of interest that we wanted to study (i.e., the presence of intracavitary effusion). On the other hand, a control group of healthy animals would have weakened the study, because different for too many aspects from a diseased group and not even representative of the general population. Selection of healthy subjects has been heavily criticized in the modern literature and the so-called “healthy patient bias” has been coined (REFERENCES follow).

1) Bloom MS, Schisterman EF, Hediger ML. Selecting controls is not selecting “normals”: design and analysis issues for studying the etiology of polycystic ovary syndrome. Fertil Steril. 2006;86(1):2-12

2) Lewallen S, Courtright P. Epidemiology in practice: case-control studies. Comm Eye Health 1998;11:57-58

3) Wacholder JK, Silverman JS, McLaughlin JK, Mandel JS. Selection of controls in case-control studies. II. Types of Controls. Am J Epidemiol; 1992, 135:1029-1041

4) Shrank WH, Patrick AR, Brookhart A. Healthy user and related biases in observational studies of preventive interventions: a primer for physicians. J Gen Intern Med, 2011;26(5):546-550

**2)** The presence of bleeding in a portion of the individuals in both groups complicates the interpretation. The authors should include an analysis of their results if these animals are excluded.

**Authors’ reply:** We thank the Reviewer for this comment that allow us to improve our article. To address your concern additional statistical analysis excluding bleedings dogs from both groups were performed as requested. The additional analyses showed that bleeding is not the solely driving of our finding. In fact, excluding these dogs none of the results changed. Please see 'Revised Manuscript with Track Changes’ at:

• Materials and methods lines: 214-220

• Results: lines 269-271; 279-283; 357-360; 364-367; 372-374; Table 1, 3, 4, and 5.

• Discussion: 427–430; 462–462; 484–485; 520–521.

6. PLOS authors have the option to publish the peer review history of their article ([what does this mean?](https://journals.plos.org/plosone/s/editorial-and-peer-review-process#loc-peer-review-history)). If published, this will include your full peer review and any attached files.


If you choose “no”, your identity will remain anonymous but your review may still be made public.


**Do you want your identity to be public for this peer review?** For information about this choice, including consent withdrawal, please see our [Privacy Policy](https://www.plos.org/privacy-policy).

Reviewer #1: No

**Authors’ reply:** none requested

Reviewer #2: No

**Authors’ reply:** none requested
