# Supplementary material for: Enhanced fibrinolysis detection in a natural occurring canine model with intracavitary effusions: Comparison and degree of agreement between thromboelastometry and FDPs, D-dimer and fibrinogen concentrations
Source: PLoS One. 2019 Nov 14;14(11):e0225089. doi: 10.1371/journal.pone.0225089 (PMC6855488; doi:10.1371/journal.pone.0225089)
Supplement: Attachment — Submitted filename: Response to Reviewers.docx [file pone.0225089.s003.docx]

**RE: Manuscript PONE-D-19-15389R1**

Enhanced fibrinolysis detection in a natural occurring canine model with intracavitary effusions: comparison and degree of agreement between thromboelastometry and FDPs, D-dimer and fibrinogen concentrations

Dear Dr. Pablo Garcia de Frutos,

The authors would like to thank You and the Reviewers for the time spent and for their constructive comments in attempt to improve our manuscript.

Few changes (listed below) have been made to the current manuscript to incorporate the suggestions of the Reviewers in the organization of the section of the manuscript. We hope that these are satisfactory to allow for publication.

Kind regards,

Andrea Zoia, DVM, Cert SAM, Dip ECVIM-CA

(Corresponding Author)

PS: If you have any further questions or comments, please do not hesitate to contact me.

**Comments to the Author**

**1.** If the authors have adequately addressed your comments raised in a previous round of review and you feel that this manuscript is now acceptable for publication, you may indicate that here to bypass the “Comments to the Author” section, enter your conflict of interest statement in the “Confidential to Editor” section, and submit your "Accept" recommendation.

Reviewer #1: (No Response)

**Authors’ reply:** none requested

Reviewer #2: All comments have been addressed

**Authors’ reply:** none requested

**2.** Is the manuscript technically sound, and do the data support the conclusions?

The manuscript must describe a technically sound piece of scientific research with data that supports the conclusions. Experiments must have been conducted rigorously, with appropriate controls, replication, and sample sizes. The conclusions must be drawn appropriately based on the data presented.

Reviewer #1: Yes

**Authors’ reply:** none requested

Reviewer #2: Yes

**Authors’ reply:** none requested

**3.** Has the statistical analysis been performed appropriately and rigorously?

Reviewer #1: Yes

**Authors’ reply:** none requested

Reviewer #2: Yes

**Authors’ reply:** none requested

**4.** Have the authors made all data underlying the findings in their manuscript fully available?

The [PLOS Data policy](http://www.plosone.org/static/policies.action#sharing) requires authors to make all data underlying the findings described in their manuscript fully available without restriction, with rare exception (please refer to the Data Availability Statement in the manuscript PDF file). The data should be provided as part of the manuscript or its supporting information, or deposited to a public repository. For example, in addition to summary statistics, the data points behind means, medians and variance measures should be available. If there are restrictions on publicly sharing data—e.g. participant privacy or use of data from a third party—those must be specified.

Reviewer #1: Yes

**Authors’ reply:** none requested

Reviewer #2: Yes

**Authors’ reply:** none requested

**5.** Is the manuscript presented in an intelligible fashion and written in standard English?

PLOS ONE does not copyedit accepted manuscripts, so the language in submitted articles must be clear, correct, and unambiguous. Any typographical or grammatical errors should be corrected at revision, so please note any specific errors here.

Reviewer #1: Yes

**Authors’ reply:** none requested

Reviewer #2: Yes

**Authors’ reply:** none requested

**6.** Review Comments to the Author

Please use the space provided to explain your answers to the questions above. You may also include additional comments for the author, including concerns about dual publication, research ethics, or publication ethics. (Please upload your review as an attachment if it exceeds 20,000 characters)

**Reviewer #1:** Thank you for mostly addressing the major concerns raised in my previous review. I have a few additional minor comments that I would recommend the authors address before publication.

(1) Thank you for better describing the rationale behind the study regarding the potential contribution of effusions to systemic hyperfibrinolysis. Given that this is the main point of the study, I would recommend that you move that description from the Discussion to the Introduction.
**Authors’ reply:** The relevant paragraphs has been moved from the discussion ('Revised Manuscript with Track Changes’ at lines: 442 - 446) to the introduction as requested ('Revised Manuscript with Track Changes’ at lines: 63 - 68)

.

(2) Your manuscript as it stands does not include any hypotheses. In the interest of informing the reader about the rationale for your statistical analyses, it would be appropriate to rewrite the final paragraph of your introduction to reframe the objectives of your study as specific, testable hypotheses that are linked back to your statistical analyses.

Best of luck with your manuscript.

**Authors’ reply:** The final paragraph has been re-written stating our 3 hypotheses of the study. i.e.:

1) ROTEM could detect an enhanced and more severe pattern of fibrinolysis in dogs with intracavitary effusions compared to dogs without effusion.
2) There was an agreement in fibrinolysis severity detection between the combination of ROTEM assay results and fibrinogen concentrations, or alternatively by concentrations of FDPs, D-dimers and fibrinogen.
3) Dogs with intracavitary effusions had more primary fibrinolysis/hyperfibrinolysis compared to dogs without effusion when assessed by concentrations of FDPs, D-dimers and fibrinogen.

Please see the 'Revised Manuscript with Track Changes’ at lines: 82 – 95.

**Reviewer #2:** The authors have answered the criticisms on the initial version of the manuscript. The laboratory reference intervals could be included in the relevant tables and figure.

**Authors’ reply:** The laboratory reference intervals have been included in the relevant tables and figure as requested. Please see 'Revised Manuscript with Track Changes’ at: table 1 and 2 + Fig 1.

**7.** PLOS authors have the option to publish the peer review history of their article ([what does this mean?](https://journals.plos.org/plosone/s/editorial-and-peer-review-process#loc-peer-review-history)). If published, this will include your full peer review and any attached files.

If you choose “no”, your identity will remain anonymous but your review may still be made public.

**Do you want your identity to be public for this peer review?** For information about this choice, including consent withdrawal, please see our [Privacy Policy](https://www.plos.org/privacy-policy).

Reviewer #1: No

**Authors’ reply:** none requested

Reviewer #2: No

**Authors’ reply:** none requested
